# Catalogue of Society Transactions

**Published:** 1890-07

**Authors:** 


CATALOGUE OF SOCIETY TRANSACTIONS.—William J. Dornan, Esq., printer, of No. 100 North Seventh street, Philadelphia, Pa., has issued a neat and convenient catalogue of the transactions of seven of the National special societies, viz., The American Gynecological Society; The American Association of Obstetricans and Gynecologists; The American Surgical Association; The Association of American Physicians; The Southern Surgical and Gynecological Association; The American Orthopedic Association, and the American Climatological Association. All the volumes issued by these several societies are printed and sold by Mr. Dornan, who is entitled to be regarded as a specialist in this class of printing. This catalogue is a complete index of these publications, and should be obtained by all interested in such literature. Send for one.

